# Exosomal miR-4800-3p Aggravates the Progression of Hepatocellular Carcinoma *via* Regulating the Hippo Signaling Pathway by Targeting STK25

**DOI:** 10.3389/fonc.2022.759864

**Published:** 2022-06-08

**Authors:** Haoming Lin, Jicai Peng, Taifeng Zhu, Meihong Xiong, Rui Zhang, Liming Lei

**Affiliations:** ^1^ Department of HBP Surgery, Sun Yat-sen Memorial Hospital, Sun Yat-sen University, Guangzhou, China; ^2^ Department of Emergency, Sun Yat-sen Memorial Hospital, Sun Yat-sen University, Guangzhou, China; ^3^ Department of Intensive Care Unit of Cardiovascular Surgery, Guangdong Cardiovascular Institute, Guangdong Provincial People’s Hospital, Guangdong Academy of Medical Sciences, Laboratory of South China Structural Heart Disease, Guangzhou, China

**Keywords:** miR-4800-3p, exosome, HCC, STK25, stemness, EMT, Hippo signaling pathway

## Abstract

**Background:**

Emerging evidence has shown that exosome microRNAs (miRNAs) regulate the development of hepatocellular carcinoma (HCC). Here, the influences of miR-4800-3p on the progression of HCC were explored.

**Materials and Methods:**

The expression of miR-4800-3p in the exosome derived by transforming growth factor beta 1 (TGF-β1)-treated HCC cells and the serum exosome isolated from HCC patients were identified by real-time PCR. The effects of TGF-β1 and the influences of Huh7-secreted exosomes and the effects of miR-4800-3p combined with/without STK25 on cell functions were explored using the EdU assay cloning experiments, wound healing assay, and Transwell assay. The corresponding molecular mechanisms were further detected using Western blot and real-time PCR assays. The combination of miR-4800-3p and STK25 was verified by the dual-luciferase and RNA pulldown assays. The influences of miR-4800-3p on the growth and epithelial–mesenchymal transformation (EMT) of implanted tumors were tested *in vivo* and further confirmed by Western blot.

**Results:**

The miR-4800-3p expression was highly expressed in both exosomes derived by TGF-β1-treated HCC cells and the serum exosomes of HCC patients. In the cases of treatment with both Huh7-derived exosomes, the level of miR-4800-3p expression was highest, and the treatment of TGF-β1 could greatly promote the proliferation, stemness, migration, and invasion of HCC cells *via* upregulating the markers of stemness and EMT, including CD44, CD133, OCT4, N-cadherin, E-cadherin, and ZO-1. Similar results could be obtained when miR-4800-3p was overexpressed in HCC cells. Furthermore, downregulation of STK25 expression, a direct target gene of miR-4800-3p, could greatly rescue the malignant biological behaviors aggravated by overexpression of miR-4800-3p. This was achieved by suppressing the expression of CD44, CD133, OCT4, N-cadherin, and PCNA and activating the Hippo pathway while increasing E-cadherin and ZO-1. Similar results were also obtained *in vivo* that knockdown of miR-4800-3p expression suppressed tumor growth induced by Huh7-derived exosomes by mediating the EMT markers and the Hippo signaling pathway.

**Conclusion:**

Exosomal miR-4800-3p could accelerate HCC development by regulating the Hippo signal by targeting STK25, which could be used as a new therapeutic target for HCC treatment.

## Introduction

Hepatocellular carcinoma (HCC), a heterogeneous disease with multiple etiologies, is the second leading cause of cancer-related death worldwide (1). Although current therapies for HCC are surgical resection, liver transplantation (LT), and interventional radiology (2–4), the high postoperative recurrence rate of HCC and the easy postoperative recurrence of intrahepatic metastases, vascular invasion, and distant organ metastases remain the greatest challenges for the treatment of HCC (2, 5). Meanwhile, due to the high degree of malignancy and rapid progression of HCC, many patients have a high postoperative recurrence rate, and the long-term efficacy is still not satisfactory (6). Therefore, it is of great significance to further study the pathogenesis and recurrence, and metastasis mechanism of HCC and search for effective molecular targets with clinical therapeutic value.

Exosomes, which are small extracellular vesicles with lipid bilayers, can be derived from various cell types, including T cells, red blood cells, and tumor cells (7, 8). Reports have shown that normal-cell-derived exosomes can transfer tumor suppressor genes to cancer cells and inhibit tumor cell growth by inhibiting the expression of oncogenes, while tumor cell-derived exosomes (Texs) can affect the local microenvironment of proximal tumor cells and stromal cells and regulate tumor neovasculogenesis, premetastatic microenvironment, etc. (8, 9). Reports also demonstrated that exosomes could carry many information molecules, such as messenger RNA (mRNA) and microRNA (miRNA), from the parent cells and deliver them to the target cells to affect the behavior of the target cells (7, 10). In HCC, exosome-carrying miRNAs are involved in the regulation of neovascularization, immune escape, epithelial–mesenchymal transformation (EMT), invasion, and multiple drug resistance (11, 12). Upregulated miR-10b and miR-21 in HCC cell-derived exosomes could increase target cell proliferation and migration by upregulating vimentin and Snail expression in target cells while decreasing the expression of the phosphatase and tensin homologue (PTEN) (13). The HCC cell-derived exosome miR-210 could be transferred to endothelial cells and target Smad4 and Stat6, thus promoting angiogenesis (14). Xiao Fu et al. showed that miR-32-5p and PTEN expressions are negatively correlated in HCC cell culture supernatants, and serum exosomes from HCC patients and high expression of miR-32-5p are positively correlated with a poor prognosis (15). Transforming growth factor β1 (TGF-β1), as an important factor in the induction of EMT *in vivo*, regulates cell growth and differentiation and promotes tumor invasion and metastasis during tumor progression (16, 17). Interestingly, TGF-β exerts both tumor-suppressive and tumor-promoting functions during cancer progression. In developing HCC, TGF-β acts as a suppressor in the early stage, but once HCC cells escape from its cyto-inhibitory effect, it leads to later tumor progression as part of its potential tumorigenic effect (16, 17). TGF-β could also induce EMT in HCC cells, thus increasing its potential for migration and invasiveness (18). It is reported that miR-4800-3p (also known as miR-4800) is differentially expressed in triple-negative breast cancer (TNBC) patients and has a poor prognosis. It is predicted that miR-4800-3p may be a biomarker for the treatment of TNBC (19). Coincidentally, Zhang et al. found that the expression of miR-4800-3p was different in colon-adenoma- and colon-cancer-related diseases, which may be used as a non-invasive screening biomarker of colon cancer (20). In addition, the miR-4800-3p is reported to be highly expressed in HCC tissues and upregulated in exosomes derived from HepG2 cells after treatment with TGF-β (21–23). However, the influences of miR-4800-3p on the progression of HCC are still unclear.

STK25, as an important member of the GCK family subgroup III (GCK III), can regulate tumor progression by inhibiting the Warburg effect, inducing apoptosis, and negatively regulating oncogene transcription (24–26). According to the Human Cancer Database, focal deletion of STK25 is common in human cancers, such as cervical squamous cell carcinoma, urinary tract carcinoma, and head and neck squamous cell carcinoma (26). In addition, in neuroblastoma, the STK25 expression level correlates with the CCM2 and TrkA expression level, and patients with high STK25 expression have a better prognosis (26). However, there is still no report on the role of STK25 in liver cancer.

Here, we explore the effect of miR-4800-3p on the cell behaviors of hepatoblastoma HepG2 cells and low-metastatic hepatoma HepB3 and LM3 cells. MiR-4800-3p expression levels in exosomes secreted by various HCC cells and serum exosomes of HCC patients and healthy people were detected. In addition, the influences of exosomes secreted from Huh7 and further the co-effects of miR-4800-3p and STK25 on the functions of HepB3 and LM3 cells were also determined. From these results, we would demonstrate for the first time the effect of miR-4800-3p of exosomes on the function of HCC cells, which could provide a new and effective target for the subsequent treatment of HCC.

## Materials and Methods

### Cell Culture

Human L-02 hepatocytes and human HCC cell lines, including Hep3B, SK-Hep-1, and hepatoblastoma HepG2, were purchased from the National Collection of Authenticated Cell Cultures (Shanghai, China). Human HCC cell line HCCLM3 (LM3) was purchased from ATCC (Manassas, VA, USA). Human HCC cell lines MHCC97H (97H) and Huh7 were purchased from BeNa Culture Collection (Beijing, China). HepG2, LM3, 97H, Huh7, and L-02 cells were cultured in Dulbecco’s modified Eagle’s medium (DMEM) (Gibco, Carlsbad, CA, USA), while Hep3B and SK cells were cultured in Eagle’s minimum essential medium (Gibco) replenished with 10% fetal bovine serum (FBS; HyClone Laboratories Inc., Novato, CA, USA) at 37°C with 5% CO_2_. In addition, the HepG2, Hep3B, and LM3 cells were treated with human recombinant TGF-β1 (10 ng/ml, R&D Systems, Minneapolis, USA) for 48 h.

### Cell Wound Healing and Invasion Assays

For the wound healing assay, HepG2, HepB3, and LM3 cells were seeded in 24-well plates (Corning, NY, USA) and cultured overnight to form a monolayer on the bottom of the plate. The straight line was scratched with the 200-μl pipette tip, and adherent cells were cultured in complete medium with TGF-β1 (10 ng/ml) for 48 h. The wound healing rate was analyzed by measuring the distance of migrated cell monolayer. Cells were imaged using a microscope (Nikon, Japan), and wound width was analyzed with Image pro plus 6.0 (Media Cybernetics, USA). For the invasion assay, a 60-μl Matrigel matrix was added to each well to evenly cover the bottom of the Transwell chamber and cultured for 60 min to a semi-solidified state. After that, 2×10^4^ HCC cells were seeded in the upper chamber. Next, a complete medium with or without TGF-β1 (10 ng/ml) was added to the 24-well plate (outer chamber). After 48 h, 4% paraformaldehyde and 0.4% crystal violet (Sigma-Aldrich, MO, USA) were used to fix and stain the cells, respectively. The number of invasive cells was measured under an inverted phase contrast microscope and calculated using ImageJ software.

### Sphere-Forming Assay

Cells were treated according to certain conditions before sphere culture with or without TGF-β1. Treated cells (1×10^2^) were seeded in ultra-low attachment 96-well plates (Corning) in serum-free DMEM/F12 (Gibco) supplement with insulin (5 μg/ml, HY-P0035, MCE), epidermal growth factor (EGF, 20 ng/ml, PeproTech), 2% B27 supplement (Invitrogen, CA, USA), and fibroblast growth factor-1 (FGF-1, 20 ng/ml, PeproTech). The cells were cultured for 7 days and then photographed using a microscope. The number of spheres (diameter > 50 μm) in each well was finally counted.

### RNA Extraction and Real-Time PCR

Total RNA was extracted from HCC cells, transfected cells, and exosomes derived from the plasma of HCC patients using TRIzol reagent (Takara, Dalian, China). Bestar™ qPCR RT kit (DBI, Germany) was used for the complementary DNA (cDNA) synthesis of mRNA. Mir-X™ miRNA First-Strand Synthesis Kit (TaKaRa) was used to synthesize the cDNA of miRNA. Bestar^®^ SYBR Green kits (DBI) were used to measure the expression of mRNA and miRNA. Real-time quantitative PCR (qPCR) was performed in Agilent Stratagene Mx3000P (Agilent, CA, USA). 2^−△△Ct^ method was used to calculate the relative expression of genes. Intracellular mRNAs and miRNAs were normalized with glyceraldehyde 3-phosphate dehydrogenase (GAPDH) and U6. Exosomal miRNAs were normalized with cel-miR-39. The related primers are listed in **Table 1**.

**Table 1:** All primers used are listed in this table.

Gene	Primer sequences
GAPDH	Forward: 5′-TGTTCGTCATGGGTGTGAAC-3′Reverse: 5′-ATGGCATGGACTGTGGTCAT-3′
STK25	Forward: 5′-CTCCGGGGATTTGCCAACC-3′Reverse:5′-TAGGTAGGAGCCAAAGTAGCG-3′
CD133	Forward: 5′-AGTCGGAAACTGGCAGATAGC-3′Reverse: 5′-GGTAGTGTTGTACTGGGCCAAT-3′
CD44	Forward: 5′-CTGCCGCTTTGCAGGTGTA-3′Reverse: 5′-CATTGTGGGCAAGGTGCTATT-3′
OCT4	Forward: 5′-CTTGAATCCCGAATGGAAAGGG-3′Reverse: 5′-GTGTATATCCCAGGGTGATCCTC-3′
E-cadherin	Forward: 5′-CGAGAGCTACACGTTCACGG-3′Reverse: 5′-GGGTGTCGAGGGAAAAATAGG-3′
N-cadherin	Forward: 5′-AGCCAACCTTAACTGAGGAGT-3′Reverse: 5′-GGCAAGTTGATTGGAGGGATG-3′
ZO-1	Forward: 5′-CAACATACAGTGACGCTTCACA-3′Reverse: 5′-CACTATTGACGTTTCCCCACTC-3′
TGF-β1	Forward: 5′-GGATACCAACTATTGCTTCAGCTCC-3′Reverse: 5′-AGGCTCCAAATATAGGGGCAGGGTC-3′
Has-miR-4800-3p	RT:5′-CTCAACTGGTGTCGTGGAGTCGGCAATTCAGTTGAGGTGGACAG-3′Forward: 5′-ACACTCCAGCTGGGCATCCGTCCGTCTGT-3′
Has-cel-miR-39	RT:5′-CTCAACTGGTGTCGTGGAGTCGGCAATTCAGTTGAGTATTACCA-3′Forward:5′-ACACTCCAGCTGGGAGCTGATTTCGTCTTGGT-3′
U6	Forward: 5’-CTCGCTTCGGCAGCACA-3’Reverse: 5′-AACGCTTCACGAATTTGCGT-3’

### Western Blotting

Radioimmunoprecipitation assay (RIPA) lysate (Beyotime, Nanjing, China) and nuclear extract kit (Active Motif, CA, USA) were used to extract the total protein from exosomes and cells, and the nuclear and cytoplasmic extracts, respectively. Protein concentration was quantitatively analyzed using a bicinchoninic acid (BCA) kit (Beyotime). The same amount of protein samples were taken from each group for electrophoresis. Then, the proteins were transferred to the polyvinylidene fluoride (PVDF) membrane and sealed with a rapid blocking solution for 30 min. The membranes were incubated with primary antibodies at 4°C overnight and then incubated with second horseradish peroxidase (HRP)-conjugated antibodies for 1 h. Finally, membranes were incubated with ECL-Plus reagent, and the blots were observed using Gel Imaging System. Related antibodies were as follows: CD44 (37259, CST, USA), CD133 (64326, CST, USA), OCT4 (2750, CST, USA), CD63 (ab134045, Abcam, USA), ALIX (92880, CST, USA), TSG101 (ab125011, Abcam, USA), Calnexin (ab133615, Abcam, USA), STK25 (ab157188, Abcam, USA), ZO-1 (8193T, CST, USA), N-cadherin (13116T, CST, USA), E-cadherin (3195T, CST, USA), p-YAP (s297) (13619, CST, USA), YAP (14074, CST, USA), TAZ (72804, CST, USA), PCNA (ab280088, Abcam, USA), GAPDH (A00227, BOSTER, China), Histone H3 (ab1791, Abcam, USA), HRP goat anti-rabbit IgG (BA1054, BOSTER, China), and HRP goat anti-mouse IgG (BA1051, BOSTER, China).

### Exosome Isolation and Identification

HCC cells were cultured in the corresponding medium with 10% exosome-depleted FBS for 48 h. The cell-conditioned medium (10 ml) or serum (250 μl, peripheral blood from HCC patients or healthy volunteers) were collected. The exosomes were isolated using ExoQuick-TC™ (System Biosciences, USA) and identified by transmission electron microscopy (TEM) and Western blot analysis, respectively. For TEM, exosomes prefixed with 4% paraformaldehyde (PFA) were loaded onto formvar carbon-coated grids and incubated for 20 min; then, the grids were refixed with 3% glutaraldehyde and 1% osmium tetroxide, respectively. After these, the exosomes were stained with uranyl oxalate, contrasted, and embedded in a mixture of 2% methylcellulose and 4% uranyl acetate with a ratio of 900 μl/100 μl, respectively. Finally, the exosomes were observed with a TEM (Hitachi 600, Hitachi, Japan). Furthermore, the expression of exosome-specific proteins CD63, TSG101, Alix, and Calnexin was detected by Western blotting analysis.

### Isolation of Exosomes From Peripheral Blood

The isolation of serum (250 μl, peripheral blood from HCC patients or healthy volunteers)-derived exosomes was conducted using the total exosome-isolation kit following the manufacturer’s recommendation. The treated serum was harvested using twice centrifugation (3,000 rpm at 4°C for 15 min; 15,000×*g* for 30 min) to remove microvesicles.

### Clinical Samples Collection

Plasma was collected from 20 HCC patients and 20 healthy volunteers who provided informed consent. The clinical information of patients is shown in **Table 2**. The miR-4800-3p expression was detected from plasma-derived exosomes. This research followed the ethical guidelines of the 1975 Declaration of Helsinki and was performed with the approval of the Sun Yat-sen Memorial Hospital, Sun Yat-sen University Institutional Review Board.

**Table 2:** Clinical information of 20 HCC patients.

Number	Gender	Age	Stage	Metastasis	Primary/secondary tumors
1	Male	52	II	–	Primary
2	Male	45	II	–	Primary
3	Female	69	II	–	Primary
4	Male	51	IIIA	–	Primary
5	Male	41	IIIB	–	Primary
6	Male	50	II	–	Primary
7	Male	58	II	–	Primary
8	Male	58	IIIB	–	Primary
9	Female	58	IIIB	–	Primary
10	Male	62	IIIB	–	Primary
11	Male	51	IB	–	Primary
12	Male	51	IB	–	Primary
13	Male	51	II	–	Primary
14	Male	51	IB	–	Primary
15	Male	62	II	–	Primary
16	Male	52	II	–	Primary
17	Male	53	IIIA	–	Primary
18	Male	68	II	–	Primary
19	Male	73	II	–	Primary
20	Female	61	II	–	Primary

### Labeling of Exosomes

The PKH67 Green Fluorescent Cell Linker Mini Kit (Sigma) was used to label exosomes. The labeled exosomes were added to HepB3 and LM3 cells in a 24-well plate. After incubation for 0.5 min, 2 h, 12 h, and 24 h, images were captured to observe the presence of exosomes within the HCC cells.

### Cell Transfection and Treatment

To change the expression of miR-4800-3p and STK25, the miR-4800-3p mimic, the miR-4800-3p inhibitor, and their negative control (NC), the overexpression of the STK25 plasmid (pcDNA3.1-STK25 plasmid, OE-STK25) and the vector plasmid pcDNA3.1 (OE-NC) was transfected into HepB3 and LM3 cells using Lipofectamine 2000 (Invitrogen, USA). The mimic, inhibitor, and miRNA-4800-3p antagomir were synthesized by Ribobio (Guangzhou, China), and the plasmids were obtained from GeneChem (Shanghai, China). Transfection efficiencies 48 h after transfection were explored by real-time PCR. In addition, to explore the effect of exosomes derived from Huh7 cells on HCC cells, HepB3 and LM3 cells were treated with exosomes (10 μg/ml) for 48 h.

### EdU Staining

The treated HepB3 and LM3 cells were labeled with 50 μM 5-ethynyl-2′-deoxyuridine (EdU; Solarbio, Beijing, China) for 2 h, and then, the cell nucleus was stained with 4′,6-diamidino-2-phenylindole (DAPI). Finally, the cells were imaged by a fluorescence microscope (Olympus, Tokyo, Japan). The proportion of EdU-positive cells in the whole visual field was analyzed.

### Immunofluorescence Assay

HepB3 and LM3 cells were fixed, permeabilized, and blocked with 1% bovine serum albumin (BSA). After these, the cells were incubated with antibodies against STK25 (ab157188, Abcam, USA) and Alexa Fluor488-conjugated goat anti-rabbit IgG (ab150077, Abcam, USA) antibody, and post-stained with DAPI. The images were photographed using fluorescence microscopy.

### Luciferase Reporter Assay

The exact mechanism of miR-4800-3p that promotes the development of HCC remains unclear. Here, the bioinformatic tools (TargetScan, StarBase, and miRDB database) were used to predict the target genes of miR-4800-3p, and the data showed that STK25 was a target gene of miR-4800-3p. Then, the luciferase reporter assay was performed to verify the combination of the untranslated region (3′-UTR) of STK25 mRNA with miR-4800-3p. HepB3 and LM3 cells were cotransfected with dual-luciferase reporter vectors [psiCHECK-2 reporter plasmids bearing STK25 3′-UTR containing wild-type (MT-STK25) or mutated (MUT-STK25) predicted miR-4800-3p binding sites] and miR-4800-3p mimics or NC. After 48 h, luciferase activities were detected using a dual-luciferase reporter assay kit (Promega, Madison, USA). Firefly luciferase activity (F) was used as the internal control.

### miRNA Target Immunoprecipitation Assay

HepB3 and LM3 cells with overexpressed miR-4800-3p or NC were collected, and the assay was performed strictly according to the procedures of the miRNA Target IP Kit (Active Motif, USA). Briefly, cells were lysed in a complete lysis buffer and then incubated with protein G-coupled magnetic beads to immunoprecipitate miRNA/mRNA complexes. Next, complexes were treated with proteinase K digestion to elute RNA from beads, and RNA was extracted using phenol:chloroform:isoamyl alcohol (25:24:1). The relative expression of STK25 was detected by qRT-PCR.

### Tumor Formation and Treatment in Nude Mice

To explore the role of exosomes and miR-4800-3p *in vivo*, 2×10^6^ LM3 cells per mouse were subcutaneously injected to establish xenografts (on the right). The mice were then randomly divided into four groups (N=5): Control, Exo, miR-4800-3p antagomir, and Exo+miR-4800-3p antagomir. Sterile saline (50 μl), exosomes (50 µg in 50 μl), and/or miR-4800-3p antagomir (20 nmol in 50 μl) were injected every 3 days for 4 weeks at 3-4 points in the subcutaneous tumors, respectively. The volume of implanted tumors was then calculated using the formula V =  a × b^2^/2 (a is the long axis, and b is the short axis). The male BALB/c nude mice (6 weeks old, 18 ± 2 g) were used in this study. Mice were sacrificed after 4 weeks of Exo and/or miR-4800-3p antagomir treatment, and the tumor tissues were removed and weighed. All animal studies were approved by the Animal Care Committee of Sun Yat-sen Memorial Hospital, Sun Yat-sen University in accordance with Institutional Animal Care and Use Committee guidelines.

### Statistical Analysis

In this study, the cell experiment had three biological repetitions, and the Transwell invasion experiment had six biological repetitions (visual field statistics). Statistical analysis was carried out using GraphPad Prism 7.0 software (GraphPad, CA, USA), and results were represented as mean ± standard deviation. Unpaired *t*-tests and one-way ANOVA were performed to compare the two and multiple groups, respectively. A *p*-value < 0.05 was defined as statistically significant.

## Results

### TGF-β1 Promotes HCC Cell Migration, Invasion, and Stemness

**Figure 1:**
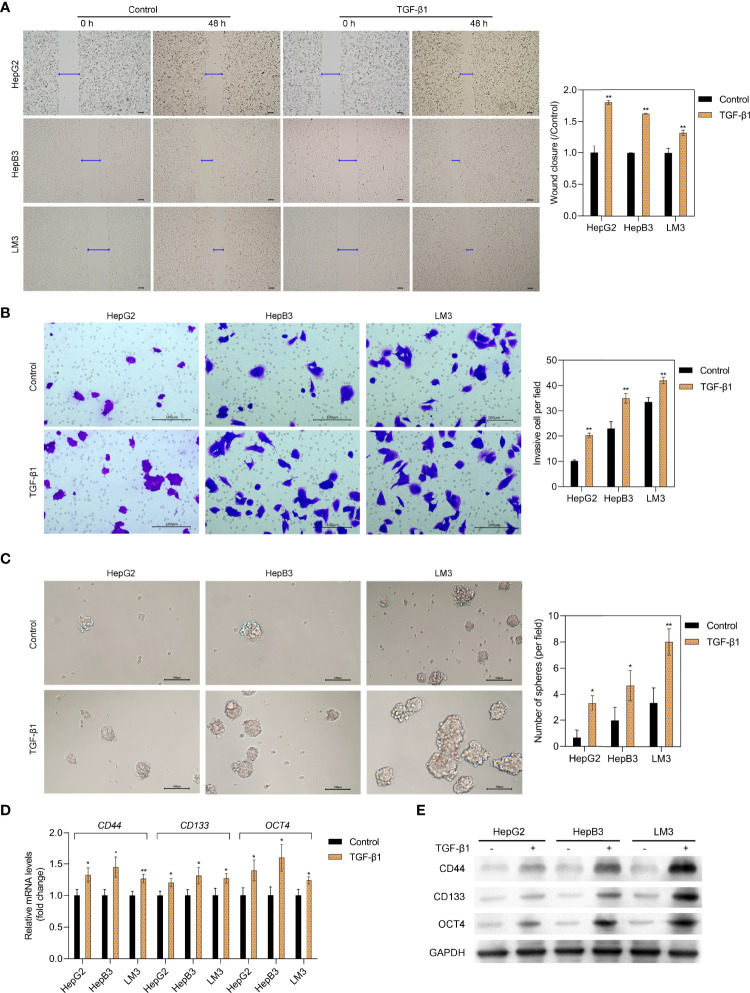
TGF-β1 promotes the migration, invasion, and stemness of HCC cells. The migration and invasive capacity of HepG2, HepB3, and LM3 cells treated with or without TGF-β1 (10 ng/ml) for 48 h was assessed by the scratch wound assay **(A)** and the Transwell assay **(B)**, respectively. Scale bar = 100 μm. **(C)** Sphere-forming assay of HepG2, HepB3, and LM3 cells treated with or without TGF-β1 (10 ng/ml). qPCR **(D)** and Western blot analysis **(E)** of tumor stem cell markers in HepG2, HepB3, and LM3 cells treated with or without TGF-β1 (10 ng/ml) for 48 h *p < 0.05, **p < 0.01 vs. control.

To explore the effect of TGF-β1 on HCC cells, HepG2 hepatoblastoma cells and low-metastatic HCC cells (HepB3 and LM3) were selected. The wound healing and Transwell assay results showed that TGF-β1 could markedly increase the migration and invasion abilities of HepG2, HepB3, and LM3 cells (**Figures 1A, B** **)**. In addition, the sphere-forming assay further confirmed that TGF-β1 could significantly promote the stemness of HepG2, HepB3, and LM3 cells (**Figure 1C**). Furthermore, we also obtained the upregulation of tumor stem cell markers (CD44, CD133, and OCT4) in HepG2, HepB3, and LM3 cells with the TGF-β1 treatment at both RNA and protein levels (**Figures 1D, E** **)**. All these data demonstrated that TGF-β1 promotes the malignant progression of HCC cells.

### Expression of miR-4800-3p in Clinical Samples and Human HCC Cells and Its Exosomes

**Figure 2:**
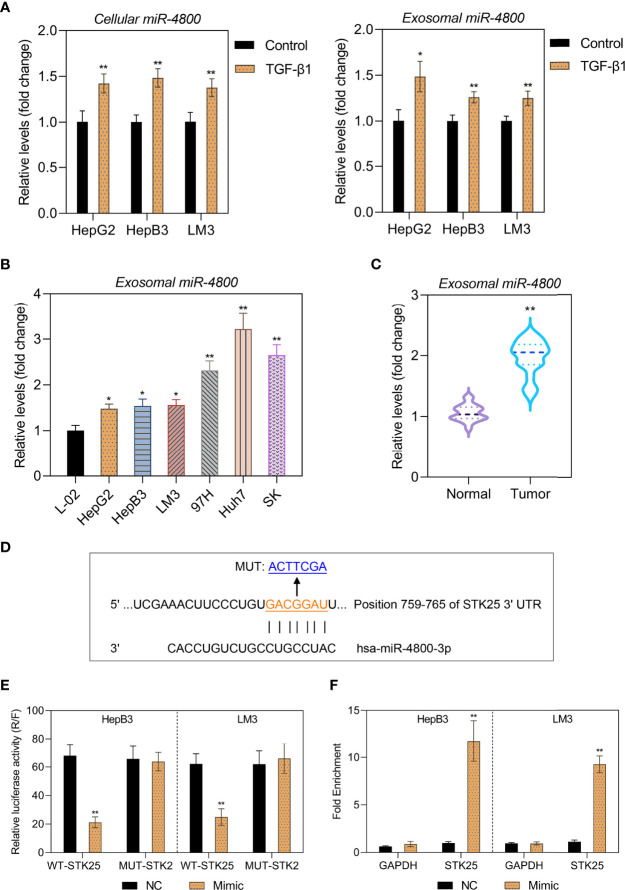
Expression of miR-4800-3p in human HCC cells and clinical samples and their exosomes. **(A)** The miR-4800-3p in HCC cells (HepG2, HepB3, and LM3) and their exosomes treated with or without TGF-β1 (10 ng/ml) for 48 h were evaluated by qPCR. **(B)** miR-4800-3p in exosomes derived from L-02 cells and HCC cells (HepG2, HepB3, LM3 97H, Huh7, and SK) was measured by qPCR assay. **(C)** The expression of exosomal miR-4800-3p derived from plasma from HCC patients or healthy volunteers was measured by a qPCR assay. **(D)** The binding sites between miR-4800-3p and STK25 in the 3′-UTR were predicted by the TargetScan and the miRDB database. **(E)** A luciferase reporter assay was performed to observe luciferase activities after transfection of the WT-STK25 or MUT-STK25 reporter together with miR-4800-3p mimic or NC in HepB3 and LM3 cells. **(F)** qPCR analysis of miR-4800-3p and STK25 incorporated into RISC in HepB3 and LM3 cells overexpressing miR-4800-3p compared to levels in the NC group. *p < 0.05, **p < 0.01 vs. control, L-02, or normal.

Exosome-encapsulated tumorigenic miRNAs could regulate tumor progression and cell communication. Here, we isolated exosomes from L-02 hepatocytes and six HCC cell lines (HepG2, HepB3, LM3, 97H, Huh7, and SK) and explored the role of related exosomal miR-4800-3p in the progression of HCC. As shown in **Supplementary Figure S1A**, exosomes were verified by TEM to have a typical cup-shaped morphology with a diameter of approximately 30–100 nm and further confirmed by Western blotting with positive exosomal markers (CD63, ALIX, and TSG101) in the exosomal fraction in both the control group and TGF-β1 group (**Supplementary Figure S1B**). Furthermore, the results also showed that, as a negative control, Calnexin was absent in the exosomal fraction (**Supplementary Figure S1B**). The expression of miR-4800-3p in HCC cells and cells-derived exosomes were further detected and markedly upregulated in TGF-β1-treated HCC cells (HepG2, HepB3, and LM3) and related cell-derived exosomes (**Figure 2A**). Furthermore, the expression of exosomal miR-4800-3p in HCC cells (HepG2, HepB3, LM3, 97H, Huh7, and SK) was higher than in L-02 hepatocytes, and the highest levels of miR-4800-3p were detected in Huh7-derived exosomes (**Figure 2B**). However, the expression of TGF-β1 demonstrated that endogenous TGF-β1 was not differentially expressed in exosomes derived from HCC cells and HCC cells (HepB3 and LM3) (**Supplementary Figure S1C**). Furthermore, higher levels of miR-4800-3p were detected in plasma exosomes from HCC patients than from healthy volunteers (**Figure 2C**). These data indicate that miR-4800 is elevated in cells and exosomes from HCC cell lines and patients.

Since our previous data showed the inverse expression pattern of STK25 and miR-4800-3p, it is still uncertain whether STK25 is a target of miR-4800-3p. To identify this prediction, the TargetScan, StarBase, and miRDB databases were used to predict the potential targets of miR-4800-3p, and the data showed the binding sites between miR-4800-3p and STK25 in 3′UTR (**Figure 2D**). The luciferase reporter plasmids containing wild-type (WT) or mutant (MUT) 3′-UTR of STK25 and cotransfected HepB3 and LM3 cells with miR-4800-3p mimic or NC mimic and WT or MUT STK25 3′-UTR were further constructed to verify the combination of STK25 and miR-4800-3p. The results showed that luciferase activity was markedly reduced when cotransfected with miR-4800-3p mimic and luc-WT-STK25 in HepB3 and LM3 cells, while luciferase activity did not change when cotransfected with miR-4800-3p mimic and luc-MUT-STK25, indicating that miR-4800-3p could directly interact with STK25 3′-UTR (**Figure 2E**). The miRNA target immunoprecipitation assay showed that STK25 was enriched in HepB3 and LM3 cells with miR-4800-3p overexpression (**Figure 2F**), further confirming that miR-4800-3p could exert its role by competitively binding STK25 to inhibit STK25 expression.

### The miR-4800-3p Promotes the Proliferation, Migration, Invasion, and Stemness in Low-Metastatic HCC Cells

**Figure 3:**
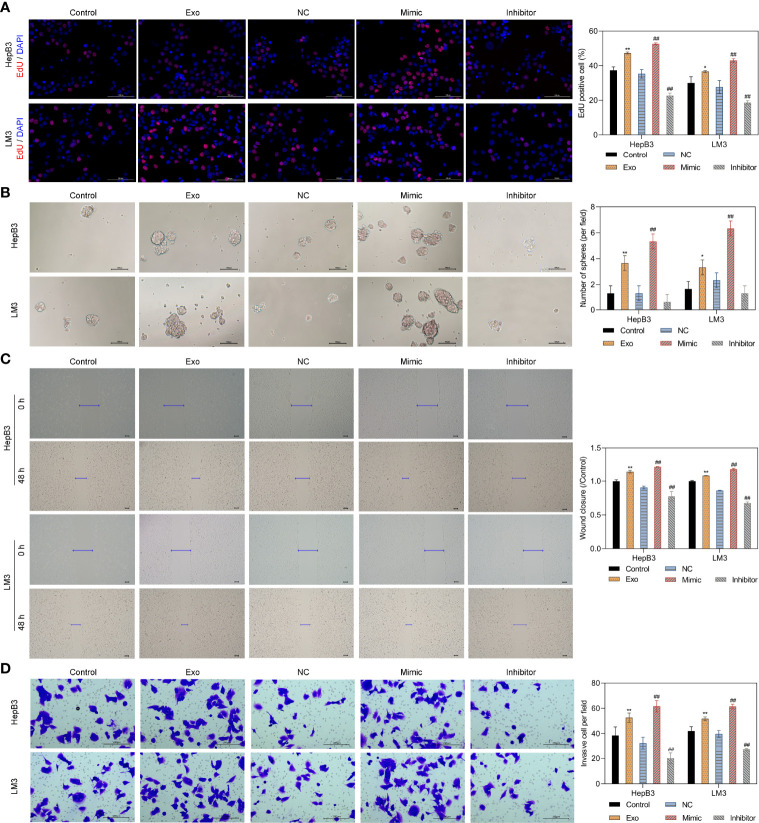
Huh7 cells with high metastatic ability transmit proliferation, stemness, migration, and invasion potential to low metastatic HCC cells through exosomal miR-4800-3p. Low-metastatic HepB3 and LM3 cells were treated with Huh7-derived exosomes, miR-4800-3p mimic, or miR-4800-3p inhibitor for 48 h, respectively. The proliferation and stemness of HepG2, HepB3, and LM3 cells were observed by EdU staining **(A)** and the sphere-forming assay **(B)**, respectively. The migration and invasive capacity of HepG2, HepB3, and LM3 cells were evaluated by the scratch wound assay **(C)** and the invasion assay **(D)**. Scale bar = 100 μm. *p < 0.05, **p < 0.01 vs. control. ^##^ *p* < 0.01 vs. NC.

As the highest levels of miR-4800-3p in HCC cell-derived exosomes, Huh7 cell-derived exosomes were selected to detect the effect of exosomal miR-4800-3p on HCC progression in subsequent experiments. To determine whether exosomal miR-4800-3p can enhance proliferation, migration, and invasion in low-metastatic HCC cells and the effects of miR-4800-3p on HCC cells, HepB3 and LM3 cells were treated with Huh7 cell-derived exosomes or transfected with miR-4800-3p mimic or miR-4800-3p inhibitor, respectively. After being treated with PKH67-labeled exosomes for 24 h, strong green fluorescent signals were detected, indicating that the recipient HCC cells (HepB3 and LM3) could successfully absorb Huh7-derived exosomes (**Supplementary Figure S2A, B**). Furthermore, after being treated with PKH67-labeled exosomes for 24 h, the expression of cellular miR-4800-3p was increased (**Supplementary Figure S2C**). The results showed that miR-4800-3p mimic promoted the expression of miR-4800-3p in exosomes, while miR-4800-3p inhibitor inhibited the expression of miR-4800-3p (**Supplementary Figure S2D**). The proliferation abilities and stemness of HCC cells were further observed by EdU staining and the sphere formation assay, respectively (**Figures 3A, B**). The results showed that exosomes derived from Huh7 with high-metastatic activity could greatly upregulate the proliferation of HepB3 and LM3 cells. Besides, the proliferation of HCC cells was enhanced when treated with miR-4800 mimics, but the knockdown of miR-4800-3p exerted an opposite effect (**Figure 3A**). Furthermore, both Huh7-derived exosomes and overexpressed miR-4800-3p could notably improve the proliferation, migration and invasion abilities, and stemness of HCC cells, while miR-4800-3p knockdown exerted an opposite effect (**Figures 3B-D**). These data indicated that high-metastatic Huh7 cells could endow low-metastatic HCC cells (HepB3 and LM3) with more powerful proliferation, migration, and metastasis abilities by secreting exosomal miR-4800-3p.

### The miR-4800-3p Promotes the Expression of Tumor Stem Cell Markers and EMT in Low-Metastatic HCC Cells by Regulating the Hippo Pathway

**Figure 4:**
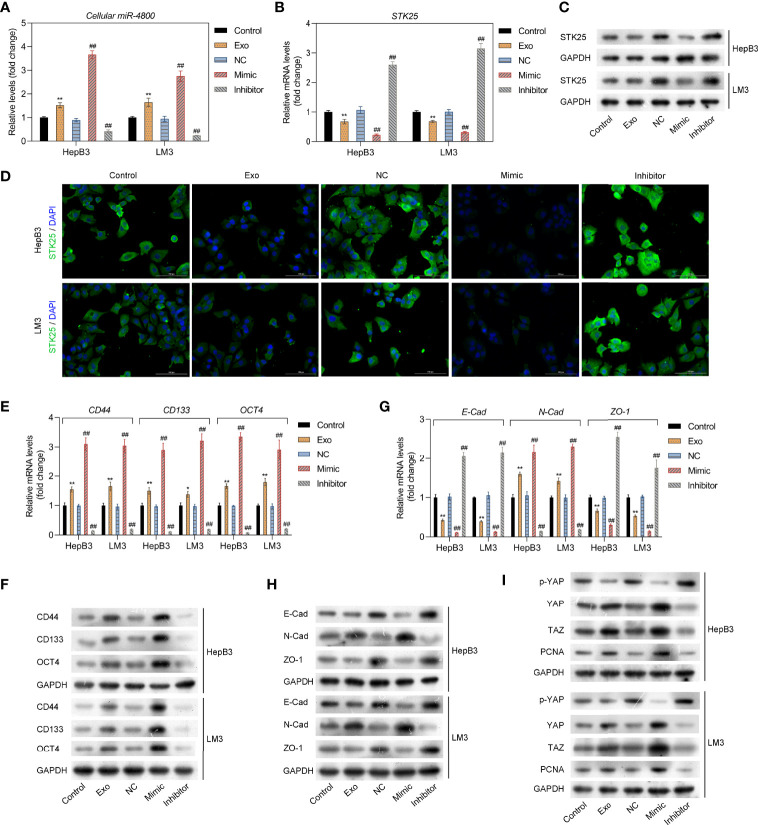
The miR-4800-3p promotes the expression of tumor stem cell markers and EMT in low-metastatic HCC cells through regulating the Hippo pathway. **(A)** The expression of miR-4800-3p was measured by qPCR assay in low-metastatic HepB3 and LM3 cells treated as previously described. STK25 expression was detected by qPCR **(B)**, Western blot analysis **(C)**, and immunofluorescence assay **(D)**, respectively. qPCR and Western blot analysis of tumor stem cell markers (CD44, CD133, and OCT4) **(E, F)** and EMT biomarkers (E-cadherin, ZO-1, N-cadherin) **(G, H)** in low-metastatic HepB3 and LM3 cells. **(I)** Western blot was used to detect the expression of p-YAP, YAP, TAZ, and PCNA in HepB3 and LM3 cells. *p < 0.05, **p < 0.01 vs. control. ^##^ *p* < 0.01 vs. NC.

To further explore the mechanism through which miR-4800-3p exhibited its role, HepB3 and LM3 were treated with Huh 7 cell-derived exosomes or transfected with miR-4800-3p mimic or miR-4800-3p inhibitor, respectively; the transfection effect and also the treatment of Huh 7 cell-derived exosomes were satisfactory (**Figure 4A**). A previous study showed that STK25 is an upstream activator of LATS kinases, whose loss notably promotes YAP/TAZ activity and enhanced cellular proliferation (25). However, it was not clear whether STK25-regulated LATS-YAP activity was involved in promoting miR-4800-3p in the malignant progression of HCC cells. Here, the expression of STK25, tumor stem cell markers, EMT, and YAP/TAZ activity in HCC cells were detected when treated with exosomal miR-4800-3p or transfected with miR-4800-3p mimic or miR-4800-3p inhibitor, respectively. Results showed that exosomal miR-4800-3p and miR-4800-3p mimic inhibited the expression of STK25 in HepB3 and LM3 cells, while miR-4800-3p inhibitor upregulated STK25 expression (**Figures 4B, C**). These results were further confirmed by immunofluorescence assay (**Figure 4D**). The expression of tumor stem cell markers (CD44, CD133, and OCT4) were also notably increased by exosomal miR-4800-3p and miR-4800-3p mimic, respectively (**Figures 4E, F**), while knockdown of miR-4800-3p decreased these tumor stem cell markers. For the detection of EMT markers, we found that exosomal miR-4800-3p or miR-4800-3p mimic treatment in HepB3 and LM3 cells could downregulate the expression of E-cadherin and ZO-1 but increase the expression of N-cadherin (**Figures 4G, H**). Similarly, opposite effects were observed when miR-4800-3p was silencing (**Figures 4G, H**). Next, it was investigated whether STK25-regulated ATS-YAP activity was involved in miR-4800-3p promoting the malignant progression of HCC cells. The activity of YAP/TAZ and the expression of proliferating cell nuclear antigen (PCNA) were explored *via* Western blot analysis. The results showed that the p-YAP in the nuclear fraction was downregulated, while the expression of YAP, TAZ, and PCNA increased in HepB3 cells under the exosomal miR-4800-3p or miR-4800-3p mimic treatment, respectively (**Figure 4I**). In contrast, silencing miR-4800-3p exerted the opposite effects (**Figure 4I**). Similar results were also obtained in LM3 cells. These data indicated that the STK25-regulates Hippo pathway participated in proliferation, migration, invasion, and EMT regulated by miR-4800-3p in HCC cells.

### The Relationship Between miR-4800-3p and STK25

**Figure 5:**
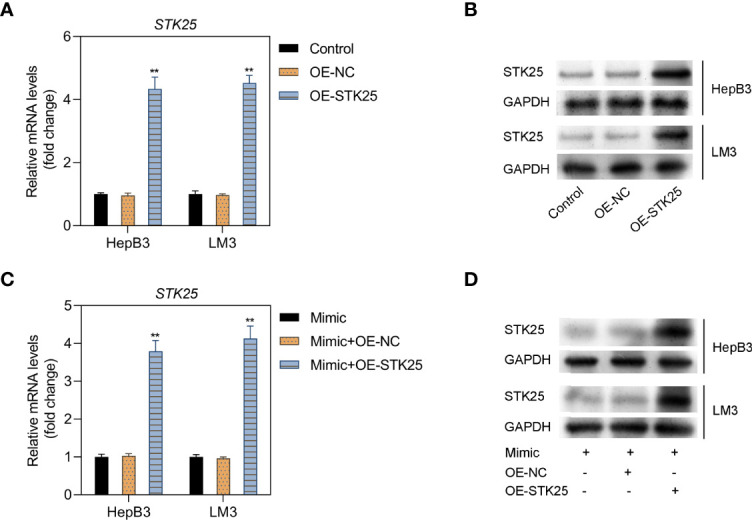
The relationship between miR-4800-3p and STK25 and detection of transfection efficiency. **(A, B)** The mRNA and protein levels of STK25 in HepB3 and LM3 cells after transfection of OE-STK25 plasmid or OE-NC plasmid. **(C, D)** mRNA and protein levels were evaluated by qRT-PCR and Western blot analysis in HepB3 and LM3 cells after transfection of OE-STK25 plasmid or the OE-NC plasmid together with the miR-4800-3p mimic. **p < 0.01.

HepB3 and LM3 cells were further cotransfected with miR-4800-3p mimic and OE-STK25 plasmid or NC plasmids, and STK25 expression increased markedly in HepB3 and LM3 cells when transfected with OE-STK25 plasmid alone (**Figures 5A, B**) or cotransfected with miR-4800-3p mimic (**Figures 5C, D**), demonstrating the combination of miR-4800-3p and STK25.

### Overexpression of STK25 Inhibits Proliferation, Invasion, and Migration of HCC Cells Promoted by miR-4800-3p

**Figure 6:**
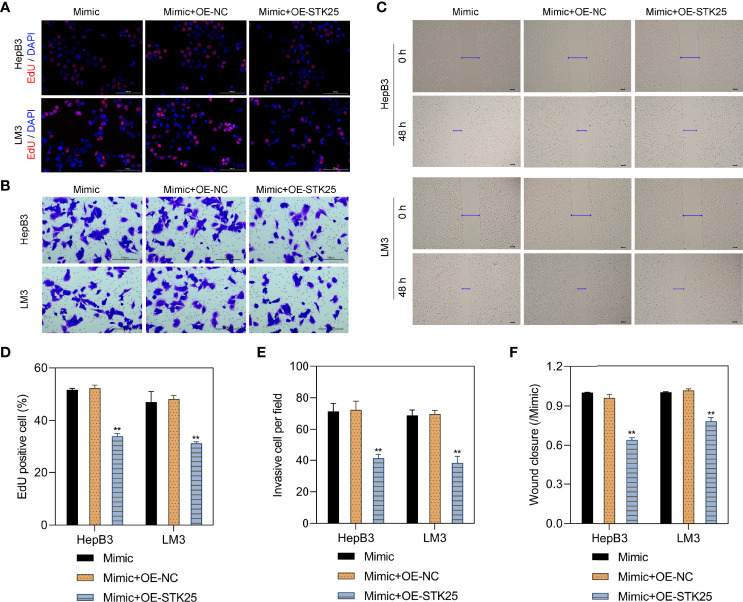
Overexpression of STK25 inhibits the proliferation, invasion, and migration of HCC cells promoted by miR-4800-3p. HepB3 and LM3 cells were transfected with the OE-STK25 plasmid or the OE-NC plasmid together with the miR-4800-3p mimic. **(A, D)** The effect of miR-4800-3p and STK25 on HepB3 and LM3 cell proliferation was evaluated by EdU staining. Scale bar, 100 μm. The effect of miR-4800-3p and STK25 on HepB3 and LM3 cell invasion and migration was observed by an invasion assay **(B, E)** and a scratch wound assay **(C, F)**. Scale bar = 100 μm. **p < 0.01 vs. mimic+OE-NC.

EdU staining showed that HepB3 and LM3 cell proliferation under miR-4800-3p mimic stimulation was markedly inhibited when treated with the OE-STK25 plasmid (**Figures 6A, D**). Invasion and wound healing assays also indicated that invasion (**Figures 6B, E**) and migration (**Figures 6C, F**) of HepB3 and LM3 cells were obviously decreased when cotransfected with the miR-4800-3p mimic and OE-STK25 plasmid. These data demonstrated that overexpression of STK25 could inhibit proliferation, invasion, and migration of HepB3 and LM3 cells.

### STK25 Overexpression Inhibits Stemness and EMT in HCC Cells Promoted by miR-4800-3p Through the Hippo Pathway

**Figure 7:**
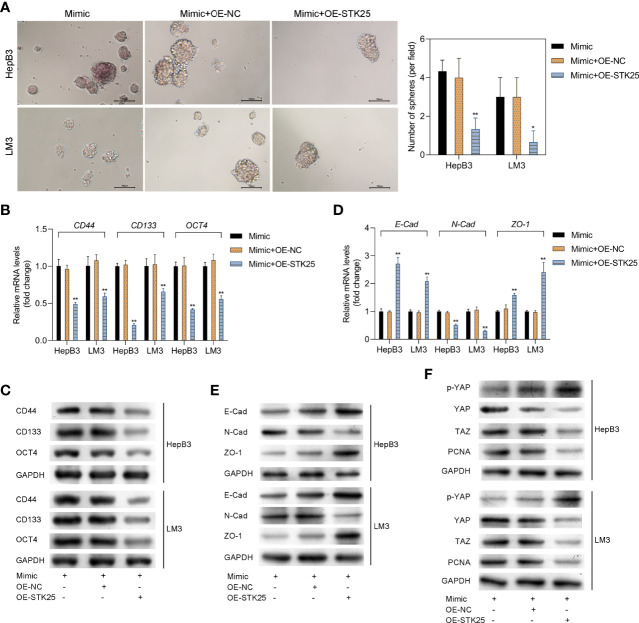
STK25 overexpression inhibits the expression of tumor stem cell markers and EMT in HCC cells promoted by miR-4800-3p through the Hippo pathway. **(A)** The stemness of HepB3 and LM3 cells was evaluated using a sphere formation assay. qPCR and Western blot analysis of tumor stem cell markers (CD44, CD133, and OCT4) **(B, C)** and EMT biomarkers (E-cadherin, ZO-1, and N-cadherin) **(D, E)** in HepB3 and LM3 cells. **(F)** Western blot was used to detect the expression of p-YAP, YAP, TAZ, and PCNA in HepB3 and LM3 cells. *p < 0.05 and **p < 0.01 vs. mimic+OE-NC.

The sphere-forming assay showed that the stemness of HepB3 and LM3 cells was markedly inhibited by overexpression of STK25 (**Figure 7A**). Correspondingly, the expression of tumor stem cell markers (CD44, CD133, and OCT4) upregulated by miR-4800-3p was also decreased by the overexpression of STK25 in HepB3 and LM3 cells (**Figures 7B, C**). Furthermore, the overexpression of STK25 could promote miR-4800-3p-suppressed E-cadherin and ZO-1 expression in HepB3 and LM3 cells but suppress miR-4800-3p-induced N-cadherin (**Figures 7D,E**). The effect of miR-4800-3p on YAP phosphorylation and YAP, TAZ, and PCNA expression were also largely reversed by the overexpression of STK25 in HepB3 and LM3 cells (**Figure 7F**). All these results indicated that the miR-4800-3p enhances malignant phenotypes of HCC cells by targeting STK25 and activating the Hippo pathway.

### Knockdown of miR-4800-3p Inhibits the Growth and EMT of Implanted Tumors Promoted by Huh7-Derived Exosomes *In Vivo*

**Figure 8:**
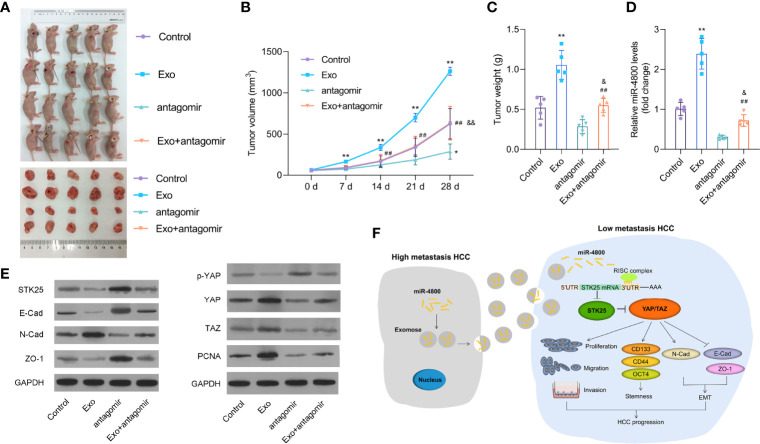
Knockdown of miR-4800-3p inhibits the growth and EMT of implanted tumors promoted by Huh7-derived exosomes *in vivo*. **(A–C)** Knockdown of miR-4800-3p suppressed tumor growth promoted by Huh7-derived exosomes. **(D)** The expression of miR-4800-3p was measured by qPCR assay. **(E)** The expression of E-cadherin, ZO-1, N-cadherin, STK25, p-YAP, YAP, TAZ, and PCNA was detected by Western blot analysis. **(F)** Schematic diagram of how exosomal miR-4800-3p is derived from high-metastatic HCC cells to promote the progression of HCC by mediating the Hippo signaling pathway by targeting STK25. Exosomal miR-4800-3p derived from high-metastatic HCC cells converted low-metastatic HCC cells to more aggressive HCC cells by mediating YAP/TAZ activation by targeting STK25 to promote stemness, proliferation, migration, invasion, and EMT in HCC. *p < 0.05, **p < 0.01 vs. control; ^##^ *p* < 0.01 vs. Exo; ^&^ *p* < 0.05, ^&&^ *p* < 0.01 vs. antagomir.

To further explore the roles of exosomes through miR-4800-3p in HCC, we built a nude mice model with LM3 cells and treated it with Huh7-derived exosomes and/or miR-4800-3p antagomir by injection. As the data revealed, miR-4800-3p antagomir treatment markedly suppressed tumor growth promoted by Huh7-derived exosomes (**Figures 8A–C**). The data showed that miR-4800-3p antagonist treatment significantly inhibited the expression of miR-4800-3p (**Figure 8D**). In addition, the EMT markers and the Hippo signaling pathway in tissues were also analyzed. As shown in **Figure 8E**, knockdown of miR-4800-3p could promote the Huh7-derived exosomes-suppressed E-cadherin and ZO-1 expression in tumor tissues but suppress the exosome-induced N-cadherin. In addition, the effect of Huh7-derived exosomes on the phosphorylation of YAP and the expression of STK25, YAP, TAZ, and PCNA were also largely reversed *via* knockdown of miR-4800-3p in HCC tumors tissues (**Figure 8E**). These results demonstrated that Huh7-derived exosomes promoted the growth and EMT of implanted tumors *in vivo* through miR-4800-3p.

## Discussion

Exosomes derived from tumor cells could promote tumor migration by creating a tumor environment or assisting tumor immune escape (8, 11, 27). As an important medium of signal communication between the tumor stroma and tumor cells, exosomal miRNAs are involved in almost the entire biological process of tumor genesis and development (10, 11). Given that serum exosomal miRNAs have high abundance, good stability, and are easy to standardize, they are often used as new biomarkers for early screening, early diagnosis, disease monitoring, and evaluation of the efficacy of HCC (9, 27). Therefore, we selected an exosomal miRNA, miR-4800-3p, which is upregulated in the serum of HCC patients and exosomes secreted by HCC cells and demonstrated that exosomes modulate the malignant phenotypes of HCC cells by delivering miR-4800-3p, which provides a new therapeutic target for HCC treatment. Hundreds of different miRNAs have been identified in exosomes released by cancer cells, and these miRNAs regulate tumor genesis and development (10). Here, we first showed that miR-4800-3p aggravated the stemness, proliferation, migration, and invasion of HCC cells and also promoted the growth of implanted tumors *in vivo*, providing effective evidence for miR-4800-3p as a new therapeutic target.

Transforming growth factor β1 (TGF-β1), as an important factor in the induction of EMT *in vivo*, regulates cell growth and differentiation and promotes tumor invasion and metastasis during tumor progression (28). Reports have shown that the TGF-β signal can promote EMT by inducing the expression of Snail/2, ZEBI/2, ET-1, OCT4, and HMGA2 (29) and also act as a tumor growth inhibitor to suppress the division and proliferation of *in situ* tumors and promote cell senescence and apoptosis in the early stage of tumors while promoting tumor cell invasion and metastasis by inducing angiogenesis and promoting immune escape in advanced stages (17). Given these effects of TGF-β1, we selected it as the inducing factor in this study. Interestingly, we proved that the expression of exosomal miR-4800 in low-metastatic HCC cells was greatly upregulated when treated with TGF-β1, while the expression of miR-4800 in high-metastatic HCC cells was also highly expressed. Thus, we speculated that the role of exosomal miR-4800 in high-metastatic HCC cells acted as TGF-β1 *via* mediating the EMT process.

HCC is a highly malignant tumor with a strong ability to invade, recur, and metastasize, which may be related to hepatocellular carcinoma cancer stem cells (HCSCs) (30). Therefore, the removal of HCSCs is a new approach for HCC treatment. In HCC, CD44 and CD133 are common cancer stem cell (CSC) markers, CD133^+^ cells have stronger proliferation ability and tumorigenicity than CD133 cells, while CD44 provides a unique cellular signature for CD133^+^ or CD90^+^ CSCs in HCC (31, 32). Octamer-binding transcription factor 4 (OCT4), a key regulatory factor of somatic reprogramming, regulates the expression of the downstream gene by binding to the promoter or enhancer of the target gene; several genes are encoded with OCT4, including sex-determining region Y-box (SOX-2) (33). Here, we show that both Huh7-derived exosomes and miR-4800-3p mimics could greatly upregulate these markers of HCSCs, indicating that miR-4800-3p could be used to treat HCC by inhibiting HCSCs and acting as an important target for HCC treatment, and also HCC-derived exosomes could be used as an effective therapeutic carrier.

Previous studies have shown that STK25 could suppress tumor cell growth and proliferation by downregulating the Golph3-dependent mTOR pathway, promoting tumor cell apoptosis by regulating CCM2TrkA, etc. (24, 34). However, as one of the important members of the GCK subfamily, STK25 has not been studied in the progression of HCC. Here, we first demonstrated the possibility of miR-4800-3p-rich exosomes as a targeted inhibitor of STK25 to achieve a similar effect in suppressing HCC progression, which is of greater importance for clinical application in HCC treatment. Therefore, we would further study STK25 expression changes in clinical samples of liver cancer and explore the related mechanism.

Reports have proven that STK25 is associated with the Hippo signal pathway (34). As the key mediators in the Hippo signal pathway, LATS1 and LATS2 kinases negatively regulate the activity of oncogene transcriptional coactivator YAP and transcriptional coactivator TAZ with PDZ binding motif (35, 36). Furthermore, a recent study has shown that the complete absence of STK25 expression often results in a partial decrease in LATS activity and an increase in YAP/TAZ activity and finally promote tumor cell proliferation (34). Considering that STK25 is a novel regulator of the Hippo signaling pathway and STK25 is a targeted gene for miR-4800-3p, we further explored the effects of miR-4800-3p on the activation of the Hippo signaling pathway and proved that overexpression of miR-4800-3p greatly reduced phosphorylated YAP in the nucleus and increased the expression of nonphosphorylated YAP/TAZ to exert the activity of transcriptional coactivators, thus promoting the transcription of downstream proliferation-related genes. Similar results were obtained from the treatment of Huh7-derived exosomes but suppressed by miR-4800-3p antagomir *in vivo*. All these results indicated that miR-4800-3p acted as an oncogenic gene by regulating the Hippo signaling pathway by targeting STK25 in both *in vitro* and *in vivo experiments*, further confirming the expression of miR-4800-3p in exosomes derived from HCC cells could also be used as a potential diagnostic marker for HCC.

Proliferating cell nuclear antigen (PCNA) is a cell cycle regulatory nuclear protein involved in DNA replication and repair (37). Its synthesis and expression can reflect the proliferation of tumor cells. A significant increase in PCNA in HCC cells often accelerates cell proliferation, and the balance between cell proliferation and apoptosis is broken (38). Here, we show that both Huh7-derived exosomes and overexpressed miR-4800-3p obviously increased PCNA expression and finally promoted HCC cell proliferation and tumor growth. These effects were largely suppressed by STK25 overexpression, confirming that miR-4800-3p aggravated HCC deterioration of HCC *via* targeting STK25.

EMT often regulates the development and progression of epithelial tumors and is closely related to tumor metastasis (39). The most important characteristic of EMT is the loss of expression of the epithelial marker E-cadherin (E-Cad) and the presence of the mesenchymal cell marker N-cadherin (N-Cad), which decrease the adhesion ability of epithelial tumor cells and improve the ability of cell migration and invasion (40). The decreased expression of E-Cad often leads to an enhanced invasion ability of tumor cells, including lung cancer cells and breast cancer cells (41). Meanwhile, previous studies have shown that zonula occludens-1 (ZO-1) is closely related to the initial formation and proliferation of tumor cells, and its abnormal expression can cause the abnormal structure and function of tight junctions and lead to invasion and metastasis of tumor cells (42). Here, we found that overexpression of miR-4800-3p could greatly suppress E-Cad and ZO-1 protein levels but increased the progression of N-Cad in both *in vitro* and *in vivo* experiments, demonstrating that miR-4800-3p could weaken the ability of invasion and metastasis of HCC cells. Interestingly, we observed a similar phenomenon in Huh7-derived exosomes, further confirming that exosomal miR-4800-3p could be used as a potential therapeutic target for HCC.

In conclusion, we proved that exosomal miR-4800-3p aggravated the progression of HCC by regulating the Hippo signaling pathway by targeting STK25 in both *in vitro* and *in vivo experiments* (**Figure 8F**), which provided evidence that exosomal miR-4800-3p could be used not only as a potential diagnostic marker for HCC but also as a therapeutic target for HCC.

## Data Availability Statement

The datasets presented in this study can be found in online repositories. The names of the repository/repositories and accession number(s) can be found in the article/**Supplementary Material**.

## Ethics Statement

This research followed the ethical guidelines of the 1975 Declaration of Helsinki and performed with the approval of the Sun Yat-sen Memorial Hospital, Sun Yat-sen University Institutional Review Board. The patients/participants provided their written informed consent to participate in this study. All animal studies were approved by the Animal Care Committee of Sun Yat-sen Memorial Hospital, Sun Yat-sen University in accordance with Institutional Animal Care and Use Committee guidelines.

## Author Contributions

HL, RZ, and LL: study design, data collection, writing/reviewing/editing manuscript, and funds collection. JP, MX, and TZ: data collection, data analysis/interpretation, and literature search. All authors contributed to the article and approved the submitted version.

## Funding

This work was supported by National Natural Science Funds of China (No. 81900285), Science and Technology Program of Guangzhou (China, No. 202002030317), and Guangdong Basic and Applied Basic Research Foundation (China, No. 2020A1515010242).

## Conflict of Interest

The authors declare that the research was conducted in the absence of any commercial or financial relationships that could be construed as a potential conflict of interest.

## Publisher’s Note

All claims expressed in this article are solely those of the authors and do not necessarily represent those of their affiliated organizations, or those of the publisher, the editors and the reviewers. Any product that may be evaluated in this article, or claim that may be made by its manufacturer, is not guaranteed or endorsed by the publisher.

## Supplementary Material

Supplementary Figure 1 Identification of exosomes derived from HCC cells and detection of the expression of TGF-β1. **(A)** Exosomes isolated from the culture supernatant of HepG2, HepB3, LM3 cells were observed by TEM. **(B)** The exosome marker proteins CD63, ALIX and TSG101 were detected by western blot analysis, and Calnexin was used as internal reference. **(C)**. The qPCR assay was performed to detect TGF-β1 expression of TGF-β1 in HCC cells (HepB3 and LM3) and exosomes derived from HCC cells.

Supplementary Figure 2 Labelling of exosomes. **(A, B)** Exosomes derived from Huh7 cells were labeled with PKH67. In addition, HepB3 and LM3 cells were incubated with labelled exosomes for 0.5 h, 2 h, 12 h and 24 h, and the images were captured using a confocal microscope. **(C)** The expression of cellular miR-4800-3p at different time points was measured by qPCR assay. **(D)** The expression of exosomal miR-4800-3p at different groups was measured by qPCR assay.

## Abbreviations

DMEM, Dulbecco’s modified Eagle’s medium; E-Cad, E-cadherin; EdU, 5-ethynyl-2’-deoxyuridine; EMT, epithelial–mesenchymal transformation; FBS, fetal bovine serum; HCC, hepatocellular carcinoma; IF, immunofluorescence; LM3, HCCLM3; MUT-STK25, mutated STK25; MT-STK25, wild-type STK25; N-Cad, N-cadherin; OE-NC, overexpression of negative control; OE-STK25, over expression of STK25; PCNA, proliferating cell nuclear antigen; PTEN, phosphatase and tensin homologue; RT-PCR, real-time PCR; SK, SK-Hep-1; TEM, transmission electron microscopy; Texs, tumor-cell-derived exosomes; ZO-1, zonula occludens-1; 97H, MHCC97H

## References

[1] Llovet JM Kelley RK Villanueva A Singal AG Pikarsky E Roayaie S. Hepatocellular Carcinoma. Nat Rev Dis Primers (2021) 7(1):6. doi: 10.1038/s41572-020-00240-3 33479224 PMC13537433

[2] Orcutt ST Anaya DA. Liver Resection and Surgical Strategies for Management of Primary Liver Cancer. Cancer Control (2018) 25(1):1073274817744621. doi: 10.1177/1073274817744621 29327594 PMC5933574

[3] Sapisochin G Bruix J. Liver Transplantation for Hepatocellular Carcinoma: Outcomes and Novel Surgical Approaches. Nat Rev Gastroenterol Hepatol (2017) 14(4):203–17. doi: 10.1038/nrgastro.2016.193 28053342

[4] Piron L Cassinotto C Guiu B. [Interventional Radiology of Liver Tumors]. Presse Med (2019) 48(10):1156–68. doi: 10.1016/j.lpm.2019.10.010 31672452

[5] Ayuso C Rimola J Vilana R Burrel M Darnell A García-Criado Á. Diagnosis and Staging of Hepatocellular Carcinoma (HCC): Current Guidelines. Eur J Radiol (2018) 101):72–81. doi: 10.1016/j.ejrad.2018.01.025 29571804

[6] Zhang X Li J Shen F Lau WY. Significance of Presence of Microvascular Invasion in Specimens Obtained After Surgical Treatment of Hepatocellular Carcinoma. J Gastroenterol Hepatol (2018) 33(2):347–54. doi: 10.1111/jgh.13843 28589639

[7] Pegtel DM Gould SJ. Exosomes. Annu Rev Biochem (2019) 88:487–514. doi: 10.1146/annurev-biochem-013118-111902 31220978

[8] Kalluri R LeBleu VS. The Biology, Function, and Biomedical Applications of Exosomes. Science (2020) 367(6478):eaau6977. doi: 10.1126/science.aau6977 32029601 PMC7717626

[9] Gilligan KE Dwyer RM. Engineering Exosomes for Cancer Therapy. Int J Mol Sci (2017) 18(6):1122. doi: 10.3390/ijms18061122 PMC5485946 28538671

[10] Pan J-H Zhou H Zhao X-X Ding H Li W Qin L. Role of Exosomes and Exosomal microRNAs in Hepatocellular Carcinoma: Potential in Diagnosis and Antitumour Treatments (Review). Int J Mol Med (2018) 41(4):1809–16. doi: 10.3892/ijmm.2018.3383 PMC5810235 29328436

[11] Wu Q Zhou L Lv D Zhu X Tang H. Exosome-Mediated Communication in the Tumor Microenvironment Contributes to Hepatocellular Carcinoma Development and Progression. J Hematol Oncol (2019) 12(1):53. doi: 10.1186/s13045-019-0739-0 31142326 PMC6542024

[12] Moris D Beal EW Chakedis J Burkhart RA Schmidt C Dillhoff M. Role of Exosomes in Treatment of Hepatocellular Carcinoma. Surg Oncol (2017) 26(3):219–28. doi: 10.1016/j.suronc.2017.04.005 28807240

[13] Tian X-P Wang C-Y Jin X-H Li M Wang F-W Huang W-J. Acidic Microenvironment Up-Regulates Exosomal miR-21 and miR-10b in Early-Stage Hepatocellular Carcinoma to Promote Cancer Cell Proliferation and Metastasis. Theranostics (2019) 9(7):1965–79. doi: 10.7150/thno.30958 PMC6485281 31037150

[14] Lin X-J Fang J-H Yang X-J Zhang C Yuan Y Zheng L. Hepatocellular Carcinoma Cell-Secreted Exosomal MicroRNA-210 Promotes Angiogenesis *In Vitro* and *In Vivo* . Mol Ther Nucleic Acids (2018) 11:243–52. doi: 10.1016/j.omtn.2018.02.014 PMC5992447 29858059

[15] Fu X Liu M Qu S Ma J Zhang Y Shi T. Exosomal microRNA-32-5p Induces Multidrug Resistance in Hepatocellular Carcinoma *via* the PI3K/Akt Pathway. J Exp Clin Cancer Res (2018) 37(1):52. doi: 10.1186/s13046-018-0677-7 29530052 PMC5846230

[16] Syed V. TGF-Beta Signaling in Cancer. J Cell Biochem (2016) 117(6):1279–87. doi: 10.1002/jcb.25496 26774024

[17] Joshi A Cao D. TGF-Beta Signaling, Tumor Microenvironment and Tumor Progression: The Butterfly Effect. Front Biosci (Landmark Ed) (2010) 15:180–94. doi: 10.2741/3614 20036814

[18] Tu S Huang W Huang C Luo Z Yan X. Contextual Regulation of TGF-Beta Signaling in Liver Cancer. Cells (2019) 8(10):1235. doi: 10.3390/cells8101235 PMC6829617 31614569

[19] Sueta A Fujiki Y Goto-Yamaguchi L Tomiguchi M Yamamoto-Ibusuki M Iwase H. Exosomal miRNA Profiles of Triple-Negative Breast Cancer in Neoadjuvant Treatment. Oncol Lett (2021) 22(6):819. doi: 10.3892/ol.2021.13080 34671433 PMC8503811

[20] Zhang Y Li M Ding Y Fan Z Zhang J Zhang H. Serum MicroRNA Profile in Patients With Colon Adenomas or Cancer. BMC Med Genomics (2017) 10(1):23. doi: 10.1186/s12920-017-0260-7 28427387 PMC5399348

[21] Huang W-J Tian X-P Bi1 S-X Zhang S-R He T-S Song L-Y. The β-Catenin/TCF-4-LINC01278-miR-1258-Smad2/3 Axis Promotes Hepatocellular Carcinoma Metastasis. Oncogene (2020) 39:4538–50. doi: 10.1038/s41388-020-1307-3 PMC7269911 32372060

[22] Zhang H Chen >X Yuan Y. Investigation of the miRNA and mRNA Coexpression Network and Their Prognostic Value in Hepatocellular Carcinoma. BioMed Res Int (2020) 2020:8726567. doi: 10.1155/2020/8726567 33274225 PMC7676931

[23] Lin H Zhang R Wu W Lei L. miR-4454 Promotes Hepatic Carcinoma Progression by Targeting Vps3A and Rab27A. Oxid Med Cell Longev (2021) 2021:923043. doi: 10.1155/2021/9230435 PMC8580624 34777698

[24] Wu F Gao P Wu W Wang Z Yang J Di J. STK25-Induced Inhibition of Aerobic Glycolysis *via* GOLPH3-mTOR Pathway Suppresses Cell Proliferation in Colorectal Cancer. J Exp Clin Cancer Res (2018) 37(1):144. doi: 10.1186/s13046-018-0808-1 29996891 PMC6042396

[25] Lim S Hermance N Mudianto T Mustaly HM Mauricio IPM Vittoria MA. Identification of the Kinase STK25 as an Upstream Activator of LATS Signaling. Nat Commun (2019) 10(1):1547. doi: 10.1038/s41467-019-09597-w 30948712 PMC6449379

[26] Costa B Kean MJ Ast V Knight JDR Mett A Levy Z. STK25 Protein Mediates TrkA and CCM2 Protein-Dependent Death in Pediatric Tumor Cells of Neural Origin. J Biol Chem (2012) 287(35):29285–9. doi: 10.1074/jbc.C112.345397 PMC3436191 22782892

[27] Han Q Zhao H Jiang Y Yin C Zhang J. HCC-Derived Exosomes: Critical Player and Target for Cancer Immune Escape. Cells (2019) 8(6):558. doi: 10.3390/cells8060558 PMC6627799 31181729

[28] Ma J Tian K Wang L Wang K Du J Li D. High Expression of TGF-Beta1 Predicting Tumor Progression in Skull Base Chordomas. World Neurosurg (2019) 131:e265–e70. doi: 10.1016/j.wneu.2019.07.128 31349076

[29] Jiang X Zhang Z Song C Deng H Yang R Zhou L. Glaucocalyxin A Reverses EMT and TGF-Beta1-Induced EMT by Inhibiting TGF-Beta1/Smad2/3 Signaling Pathway in Osteosarcoma. Chem Biol Interact (2019) 307:158–66. doi: 10.1016/j.cbi.2019.05.005 31059706

[30] Mani SKK Andrisani O. Hepatitis B Virus-Associated Hepatocellular Carcinoma and Hepatic Cancer Stem Cells. Genes (Basel) (2018) 9(3):137. doi: 10.3390/genes9030137 PMC5867858 29498629

[31] Gurzu S Kobori L Fodor D Jung I. Epithelial Mesenchymal and Endothelial Mesenchymal Transitions in Hepatocellular Carcinoma: A Review. BioMed Res Int (2019) 2019:2962580. doi: 10.1155/2019/2962580 31781608 PMC6855070

[32] Liu Y-C Yeh C-T Lin K-H. Cancer Stem Cell Functions in Hepatocellular Carcinoma and Comprehensive Therapeutic Strategies. Cells (2020) 9(6):1331. doi: 10.3390/cells9061331 PMC7349579 32466488

[33] Upadhyay VA Shah KA Makwana DP Raval AP Shah FD Rawal RM. Putative Stemness Markers Octamer-Binding Transcription Factor 4, Sex-Determining Region Y-Box 2, and NANOG in Non-Small Cell Lung Carcinoma: A Clinicopathological Association. J Cancer Res Ther (2020) 16(4):804–10. doi: 10.4103/jcrt.JCRT_213_18 32930122

[34] Bae SJ Ni L Luo X. STK25 Suppresses Hippo Signaling by Regulating SAV1-STRIPAK Antagonism. Elife (2020) 9:e54863. doi: 10.7554/eLife.54863 32292165 PMC7182433

[35] Ma S Meng Z Chen R Guan K-L. The Hippo Pathway: Biology and Pathophysiology. Annu Rev Biochem (2019) 88:577–604. doi: 10.1146/annurev-biochem-013118-111829 30566373

[36] Park JAe Kwon Y-G. Hippo-YAP/TAZ Signaling in Angiogenesis. BMB Rep (2018) 51(3):157–62. doi: 10.5483/BMBRep.2018.51.3.016 PMC5882223 29366443

[37] Sheng C Mendler I-H Sara Rieke PS Jentsch M Friedrich D Drossel B. PCNA-Mediated Degradation of P21 Coordinates the DNA Damage Response and Cell Cycle Regulation in Individual Cells. Cell Rep (2019) 27(1):48–58.e7. doi: 10.1016/j.celrep.2019.03.031 30943414

[38] Cheng H Cao X Min X Zhang X Kong Q Mao Q. Heat-Shock Protein A12A is a Novel PCNA-Binding Protein and Promotes Hepatocellular Carcinoma Growth. FEBS J (2020) 287(24):5464–77. doi: 10.1111/febs.15276 32128976

[39] Bakir B Chiarella AM Pitarresi JR Rustgi AK. EMT, MET, Plasticity, and Tumor Metastasis. Trends Cell Biol (2020) 30(10):764–76. doi: 10.1016/j.tcb.2020.07.003 PMC7647095 32800658

[40] Lv W Wang J Zhang S. Effects of Cisatracurium on Epithelial-to-Mesenchymal Transition in Esophageal Squamous Cell Carcinoma. Oncol Lett (2019) 18(5):5325–31. doi: 10.3892/ol.2019.10859 PMC6781646 31612042

[41] Li LI Lv Y Zhang Y He L Zhang H. Expression and Clinical Significance of Oct-4 and E-Cad in non-Small-Cell Lung Cancer. Oncol Lett (2016) 11(1):234–6. doi: 10.3892/ol.2015.3856 PMC4727047 26870194

[42] Kang L Shen L Lu L Wang D Zhao Y Chen C. Asparaginyl Endopeptidase Induces Endothelial Permeability and Tumor Metastasis *via* Downregulating Zonula Occludens Protein ZO-1. Biochim Biophys Acta Mol Basis Dis (2019) 1865(9):2267–75. doi: 10.1016/j.bbadis.2019.05.003 31096007

