# Supplementary figures and images for: Exosomal miR-4800-3p Aggravates the Progression of Hepatocellular Carcinoma via Regulating the Hippo Signaling Pathway by Targeting STK25
Source: Front Oncol. 2022 Jun 8;12:759864. doi: 10.3389/fonc.2022.759864 (PMC9214204; doi:10.3389/fonc.2022.759864)

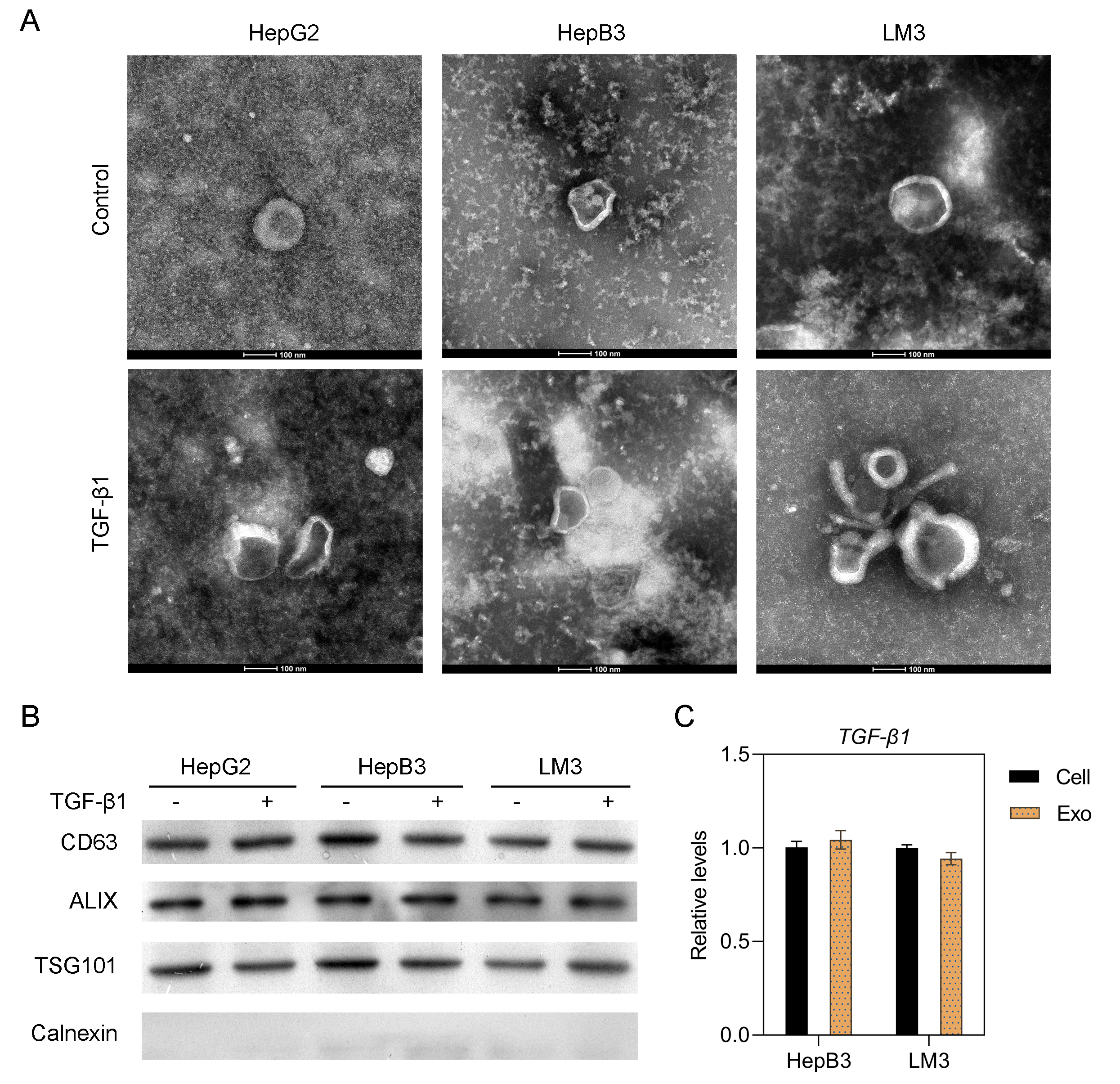

Supplement: Supplementary Figure 1 — Identification of exosomes derived from HCC cells and detection of the expression of TGF-β1. (A) Exosomes isolated from the culture supernatant of HepG2, HepB3, LM3 cells were observed by TEM. (B) The exosome marker proteins CD63, ALIX and TSG101 were detected by western blot analysis, and Calnexin was used as internal reference. (C). The qPCR assay was performed to detect TGF-β1 expression of TGF-β1 in HCC cells (HepB3 and LM3) and exosomes derived from HCC cells. [file Image_1.tif]

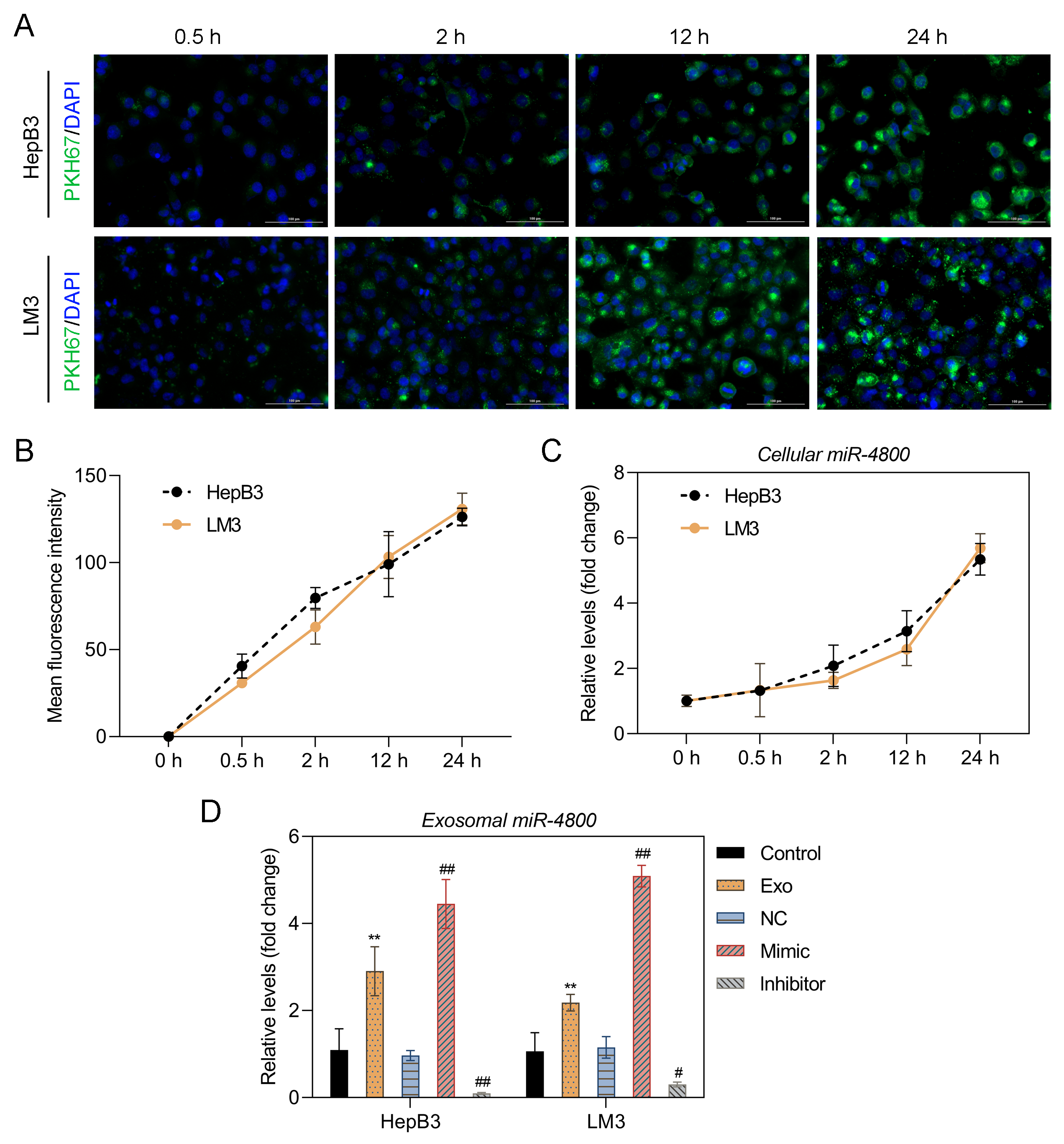

Supplement: Supplementary Figure 2 — Labelling of exosomes. (A, B) Exosomes derived from Huh7 cells were labeled with PKH67. In addition, HepB3 and LM3 cells were incubated with labelled exosomes for 0.5 h, 2 h, 12 h and 24 h, and the images were captured using a confocal microscope. (C) The expression of cellular miR-4800-3p at different time points was measured by qPCR assay. (D) The expression of exosomal miR-4800-3p at different groups was measured by qPCR assay. [file Image_2.tif]
